# Spatial distribution of preantral follicles in the equine ovary

**DOI:** 10.1371/journal.pone.0198108

**Published:** 2018-06-13

**Authors:** Benner G. Alves, Kele A. Alves, Gustavo D. A. Gastal, Melba O. Gastal, José R. Figueiredo, Eduardo L. Gastal

**Affiliations:** 1 Department of Animal Science, Food and Nutrition, Southern Illinois University, Carbondale, Illinois, United States of America; 2 Laboratory of Manipulation of Oocytes and Preantral Follicles (LAMOFOPA), State University of Ceará, Fortaleza, CE, Brazil; University of Florida, UNITED STATES

## Abstract

Comprehensive studies on spatial distribution of preantral follicles in the ovary are scarce. Considering that preantral follicles represent the main ovarian reserve, harvesting of these follicles is crucial for the development/use of assisted reproductive techniques. Therefore, knowledge on follicle spatial distribution can be helpful for targeting areas with richer number of preantral follicles through biopsy procedures. The aim of this study was to assess the distribution and localization of equine preantral follicles according to: (i) age, (ii) ovarian portion (lateral and intermediary) and region (dorsal and ventral), (iii) distance from the geometric center, and (iv) follicular class. Ovaries from young and old mares (n = 8) were harvested in a slaughterhouse and submitted to histological processing for further evaluation. For data analyses, a novel methodology was developed according to the geometric center of each histological section for a precise determination of preantral follicle distribution. Results indicated that (i) equine preantral follicles are clustered and located near to the ovarian geometric center, and that aging induced their dispersion through the ovarian cortex; (ii) the distance from the geometric center was shorter for developing follicles than primordial; and (iii) secondary follicles were more distant from the geometric center but closer to the ovulation fossa. In conclusion, the spatial distribution of preantral follicles was successfully determined in the equine ovary and was affected by age, region, and portion.

## Introduction

During the reproductive lifespan, a rich and finite number of gametes (primordial follicles) are available and represent the main oocyte ovarian reserve of an individual. Early folliculogenesis *in vivo* is a complex dynamic process featured by follicular quiescence [1], activation and growth [2], follicular migration [3], and cell interactions [4]. Once these events occur simultaneously in the ovarian parenchyma [5], studies related to preantral follicle spatial distribution can potentially help to clarify important anatomical-physiological mechanisms involved in folliculogenesis.

The equine ovary has a peculiar architecture among mammals due to the inverted arrangement of the cortical and medullary zones and presence of ovulation fossa [6,7]. Histological studies of the equine ovary have produced limited information regarding the distribution and localization of preantral follicles in the ovarian parenchyma [8–12]. Although several recent studies in mares using ovarian biopsy fragments have advanced our knowledge on preantral follicle population and features [13–16], these reports have not allowed us to determine the spatial distribution of preantral follicles in the whole ovary.

Studies in several species (women [17]; cow [18]; sheep [19]; mice [20]), including the mare [10], have shown that preantral follicles are not homogenously distributed throughout the ovarian parenchyma, and that the preantral follicle population and distribution can be influenced by several factors (for review see [21]), including age (woman [22]; cow [23]; mare [24]). As a result of the lack of information regarding preantral follicle distribution, the ovarian biopsy procedure has been done randomly in several species. This fact has resulted in harvesting of ovarian tissue with an extremely variable number of preantral follicles per fragment (mouse [25]; woman [26]; mare [16]; sheep [19]; goat [27]), which can potentially affect the efficiency of assisted reproductive techniques (ARTs), such as cryopreservation and/or transplant. Therefore, comprehensive studies about preantral follicle distribution in the ovary can be helpful for targeting areas with greater numbers of follicles, improving efficiency of the biopsy procedure.

The aims of this study were to assess the distribution and localization of equine preantral follicles according to: (i) age, (ii) ovarian portion (lateral and intermediary) and region (dorsal and ventral), (iii) distance from the geometric center, and (iv) follicular class.

## Materials and methods

### Ovaries

**Fig 1:**
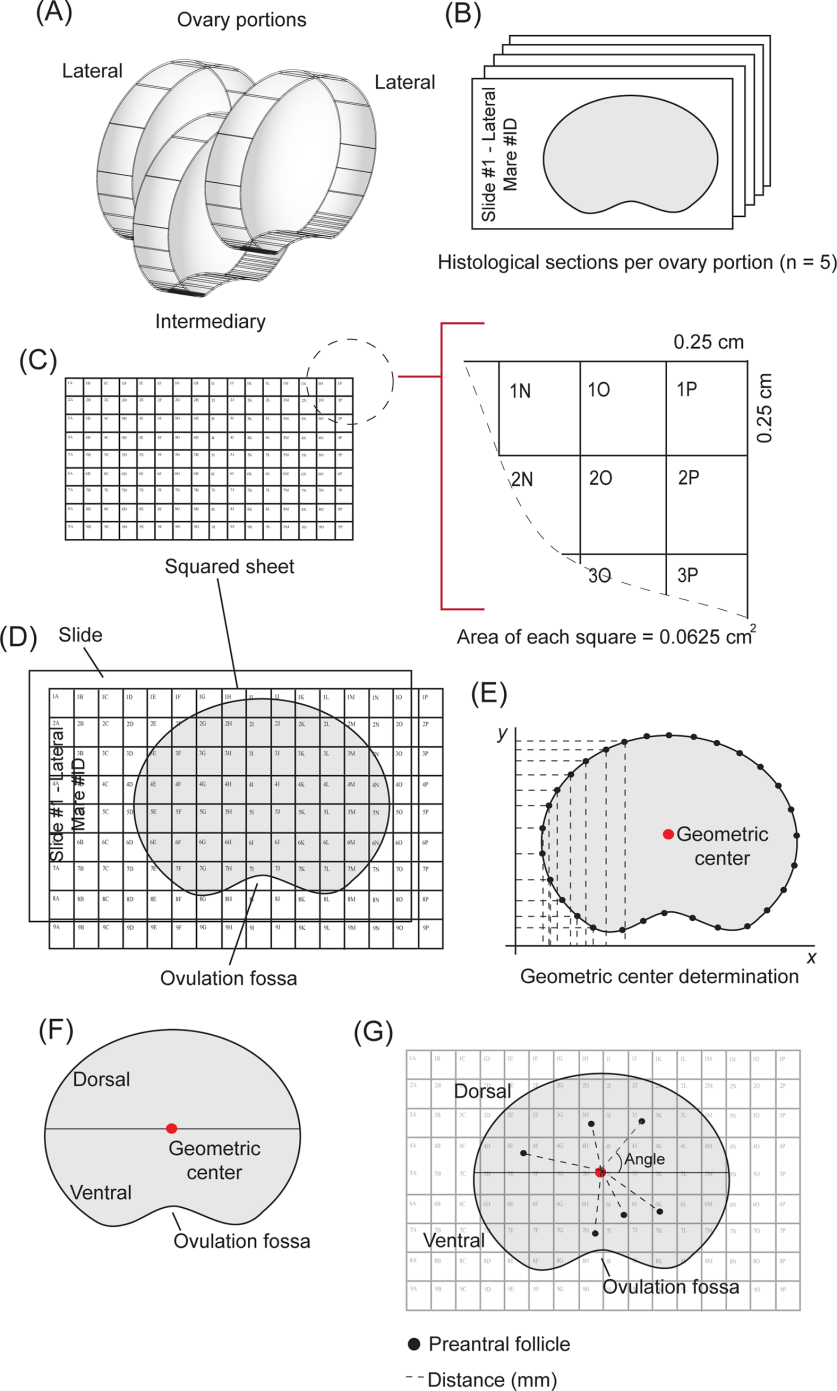
Illustration of experimental procedures performed to assess the preantral follicle spatial distribution in the equine ovary. The ovaries were divided into (A) three portions (lateral, n = 2; intermediary, n = 1), followed by (B) histological processing. Thereafter, the main steps to guide the microscopy evaluation were performed: (C) squared sheet developed with columns and rows represented by letters and numbers, respectively; (D) microscopy slides scanned jointly with squared sheet by a photo editing program; (E) geometric center determination for each histological section; (F) definition of dorsal and ventral ovarian regions; and (G) analysis of preantral follicle distribution using the squared sheet at the moment of microscope evaluation.

Ovaries from mixed-breed mares (n = 8) were harvested at an equine slaughterhouse and separated into two age groups according to dental and physical characteristics: 4˗9 years (n = 4) and >20 years (n = 4). Immediately after slaughter, each ovary was divided longitudinally into three portions designated as lateral (n = 2 portions) and intermediary (n = 1 portion) (Fig 1A). Each ovarian portion was fixed in paraformaldehyde 4% (24 hours) and placed in 70% alcohol until histological processing. Ovaries of the eight selected mares did not contain visible preovulatory follicles and/or corpus luteum, and the reproductive status (cycling or anestrus) of the mares was unknown.

### Histological processing

Ovarian portions of each mare were dehydrated, embedded in paraffin wax and totally cut into serial sections (7 μm; [15]). Considering the frequency and diameter [15] of both primordial (61%; ~33 μm) and transitional (25%; ~36 μm) follicles, respectively in the equine ovary, and to avoid follicle double counting, every 5th section was mounted on large microscope slides (127 × 102 mm). To ensure a good tissue quality for the analyses, only ovarian histological sections with clearly visible borders and intact ovulation fossa without laceration were chosen. Thus, five representative histological sections were assessed per ovarian portion (Fig 1B). Samples were stained with Periodic Acid-Schiff (PAS) and counterstained with hematoxylin.

### Steps to assess preantral follicle spatial distribution

The assessment of preantral follicle spatial distribution in the assigned ovarian portions and regions was performed as follows:

1. A squared sheet (area of each square = 0.0625 cm^2^) with columns (represented by letters) and rows (represented by numbers) was designed and printed in overhead transparency paper at the same size of the microscope slide (Fig 1C).
2. Histological sections were overlapped with the squared sheet and scanned by a photo editing program (Adobe Photoshop CS4; San Jose, USA). All histological sections were scanned with the ovulation fossa positioned at the bottom and the squared sheet aligned to the upper left corner of the slide (Fig 1D). Thus, these digitalized images, herein called “ovarian maps,” were used as locating guides for the microscopic evaluation of the preantral follicles spatial distribution.
3. The geometric center of each digitalized histological section was defined (Fig 1E). Briefly, thirty equidistant points throughout the histological section perimeter were determined (Adobe Photoshop CS4). Then, the distance of each point relative to the X and Y axes was recorded and the geometric center was calculated by the following formulas:
  $$\begin{array}{l} Mean\;distance\;X=\sum_{i=1}^{30}xi=\left(x1+x2+\ldots +x30\right)/number\;of\;points\;recorded\\ Mean\;distance\;Y=\sum_{i=1}^{30}yi=\left(y1+y2+\ldots +y30\right)/number\;of\;points\;recorded\\ Geometric\;center=mean\;distance\;\left(X;\;Y\right) \end{array}$$
4. After the geometric center was determined, a longitudinally line was made with a marker tool (Adobe Photoshop CS4) and the ovarian regions above and below (towards the ovulation fossa) the longitudinal line were termed dorsal and ventral regions, respectively (Fig 1F).
5. Finally, the distance (mm) and angle (0º˗360º) (Fig 1G) of the preantral follicles were measured into each ovarian map in relation to the geometric center with a ruler tool by the image software (Adobe Photoshop CS4).

### Microscopy and end points

The histological sections were analyzed using light microscopy (Nikon, Japan) at magnification ×400. For each ovarian portion, the following end points were evaluated: number of follicles, follicle class, and follicle spatial distribution taking into consideration the ovarian region, angle, and geometric center. Only preantral follicles with visualized oocyte nucleus were counted and classified according to their developmental stage into primordial, transitional, primary, and secondary as previously described [10]. Counting and classification of preantral follicles were performed by K. A. Alves, and follicle spatial distribution calculations by B. G. Alves.

### Statistical analyses

All statistical analyses were performed using R statistical software, version 3.0.2 (R Foundation for Statistical Computing, Vienna, Austria). Data for end points that were not normally distributed (Kolmogorov-Smirnov) were transformed in base 10 logarithm (Log10). When appropriate, t-Test and Wilcoxon–Mann–Whitney test were used to compare mean values between groups. The preantral follicle distribution according to the ovarian portion, region, and geometric center was analyzed by chi-square test. A linear regression analysis was performed to determine the relationship of age and distance of preantral follicles to the ovarian geometric center. Data are presented as mean (± SEM) and percentage, and the statistical significance was defined as *P* < 0.05 (two-sided).

## Results

Young and old mares had similar (P > 0.05) ovary measurements (length, 48.8 ± 3.5 vs. 48.6 ± 3.3 mm; height, 35.7 ± 2.4 vs. 30.7 ± 2.4 mm; and width, 31.1 ± 2.5 vs. 28.0 ± 2.2, respectively). A total of 240 ovarian maps were evaluated (15 per ovary) and 9,284 preantral follicles were recorded in lateral (n = 4,901) and intermediary (n = 4,383) ovarian portions. The frequency distribution of preantral follicles on ovarian portions and regions (dorsal and ventral) is shown (Table 1). Overall, 58% of the total number of preantral follicles were observed on the ovarian dorsal region. Ovarian portions within the same region had a similar (*P* < 0.05) frequency of preantral follicles.

**Table 1:** Frequency distribution of equine preantral follicles on ovarian portions (lateral and intermediary) and regions (dorsal and ventral).

	Frequency distribution of preantral follicles (%)		
	Ovarian portion^§^		
Ovarian region^‡^	Lateral	Intermediary	Overall
Dorsal	57.9 (2,839/4,901)^a^	57.6 (2,523/4,383)^a^	57.8 (5,362/9,284)
Ventral	42.1 (2,062/4,901)^a^	42.4 (1,860/4,383)^a^	42.2 (3,922/9,284)
Overall	52.8 (4,901/9,284)	47.2 (4,383/9,284)	-

^§^ The ovaries were divided longitudinally in three portions: two laterals (combined) and one intermediary. Both ovaries (left and right) from each mare (n = 8) were used.

^‡^ Ovarian regions were defined as dorsal (above) and ventral (below) according to the geometric center and ovulation fossa.

^a^ Within a row, no difference was observed between ovarian portions (P > 0.05).

**Fig 2:**
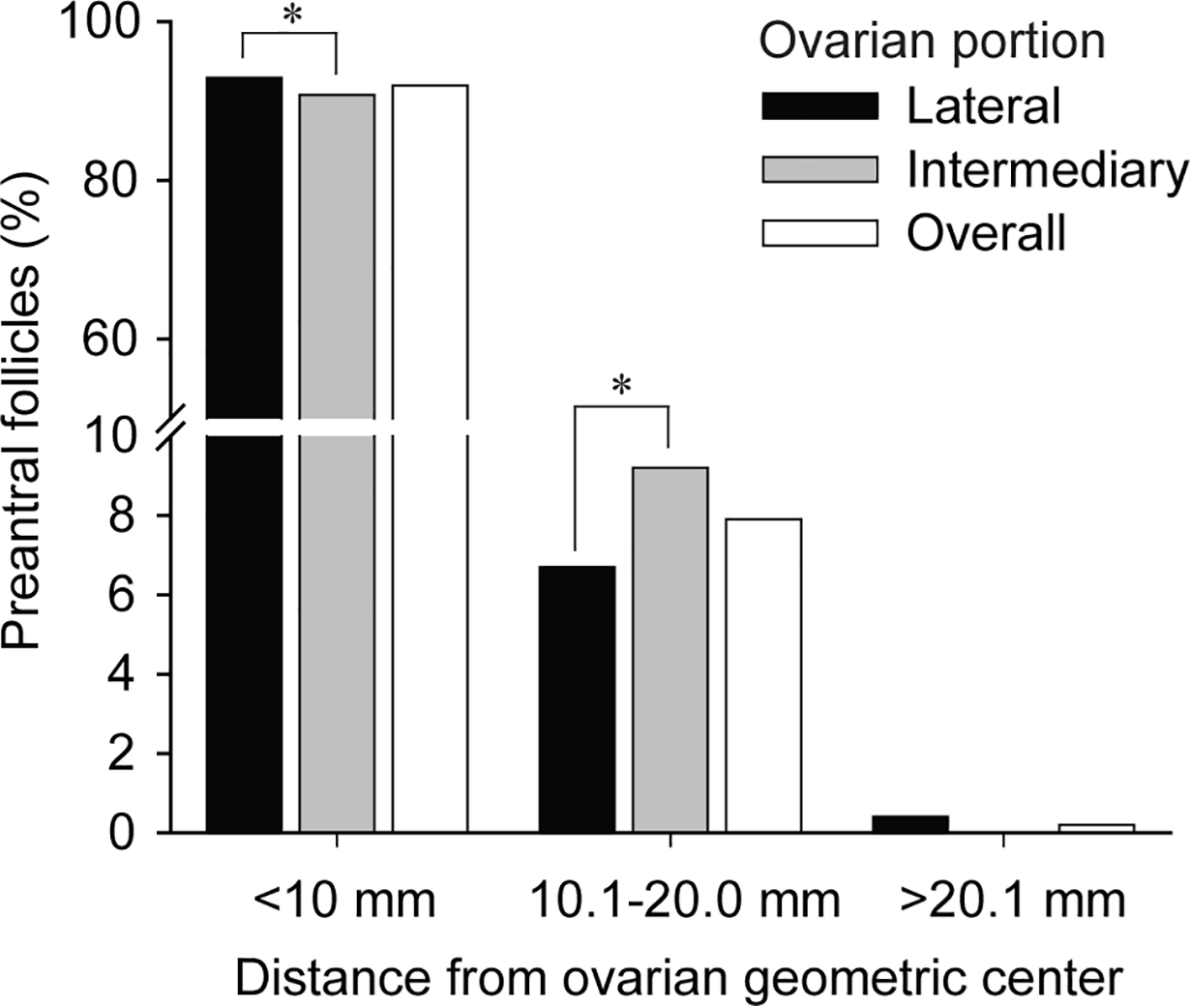
Frequency distribution of equine preantral follicles according to the geometric center in different ovarian portions (lateral and intermediary). Total number of preantral follicles evaluated per ovarian portion: lateral, n = 4,901, and intermediary, n = 4,383. * Differ between ovarian portions within the same distance interval (*P* < 0.05).

The frequency distribution of preantral follicles to the ovarian geometric center was evaluated at distance intervals (Fig 2). Regardless of ovarian portion, a greater proportion (~92%) of preantral follicles was found in the circle of 10 mm radius from the geometric center. Also, within the same distance (<10 mm) from the geometric center, the lateral portion had a greater (*P* < 0.05) number of preantral follicles compared to the intermediary portion. However, in the circle of 10.1 to 20.0 mm radius, a greater proportion (*P* < 0.05) of preantral follicles was seen in the intermediary portion.

An age effect was observed on the distance intervals from preantral follicles to the ovarian geometric center (Table 2). Young mares (4˗9 years old) had a greater (*P* < 0.05; ~95%) proportion of follicles within the circle interval of <10 mm radius compared to old mares (>20 years; ~54%), regardless of ovarian portion. In contrast, in old mares (>20 years), a greater proportion (*P* < 0.05) of preantral follicles was observed within the circle interval of 10.1˗20 mm radius in both lateral and intermediary ovarian portions.

**Table 2:** Age effect on frequency distribution of equine preantral follicles according to the geometric center in different ovarian portions (lateral and intermediary).

	Frequency distributions of preantral follicles (%)					
	Distance intervals to ovarian geometric center					
	<10.0 mm		10.1–20.0 mm		>20.1 mm	
	Age group (years)					
Ovarian portion^§^	4–9	>20	4–9	>20	4–9	>20
Lateral (n = 4,901)^†^	94.0^a^^A^	64.4^B^^a^	5.6^a^^A^	34.5^b^^A^	0.4^a^	1.1^a^
Intermediary (n = 4,383)	95.3^a^^B^	49.7^b^^B^	4.7^a^^B^	50.3^b^^B^	-	-
Overall	94.6^a^	53.9^b^	5.2^a^	45.8^b^	0.2^a^	0.3^a^

^§^ The ovaries were divided longitudinally into three portions: two laterals (combined) and one intermediary. Both ovaries (left and right) from each mare (n = 8) were used.

^†^ Number of preantral follicles evaluated.

^a,b^ Within the same distance interval and between age groups, values without a common superscript differed (*P* < 0.05).

^A,B^ Within a column, values without a common superscript differed (*P* < 0.05).

The mean distance of equine preantral follicles to the geometric center in different ovarian portions and regions was assessed (Table 3). Overall, preantral follicles observed in the ventral region were farther (*P* < 0.05) from the ovarian geometric center than those in the dorsal region. Preantral follicles in the intermediary portion were farther (*P* < 0.05) from the ovarian geometric center compared with the lateral portion.

**Table 3:** Mean (± SEM) distance from equine preantral follicles to the geometric center according to different ovarian portions (lateral and intermediary) and regions (dorsal and ventral).

	Distance from preantral follicles to ovarian geometric center (mm)		
	Ovarian portion^§^		
Ovarian region^‡^	Lateral (n = 4,901)^†^	Intermediary (n = 4,383)	Overall (n = 9,284)
Dorsal	5.51 ± 0.05^a^^A^	5.11 ± 0.05^b^^A^	5.32 ± 0.03^A^
	(0.6–23.0) ^¥^	(0.3–18.9)	(0.3–23.0)
Ventral	5.66 ± 0.07^a^^B^	7.06 ± 0.07^b^^B^	6.32 ± 0.05^B^
	(0.4–25.7)	(0.6–19.7)	(0.4–25.7)
Overall	5.58 ± 0.04^a^	5.93 ± 0.04^b^	5.74 ± 0.03
	(0.4–25.7)	(0.3–19.7)	(0.3–25.7)

^§^ The ovaries were divided longitudinally into three portions: two laterals and one intermediary. Both ovaries (left and right) from each mare (n = 8) were used.

^‡^ Ovarian regions were defined as dorsal (above) and ventral (below) according to the geometric center and ovulation fossa.

^†^ Number of preantral follicles evaluated.

^¥^ Range of follicles.

^a,b^ Within a row, values without a common superscript differed (*P* < 0.05).

^A,B^ Within a column, values without a common superscript differed (*P* < 0.05).

**Fig 3:**
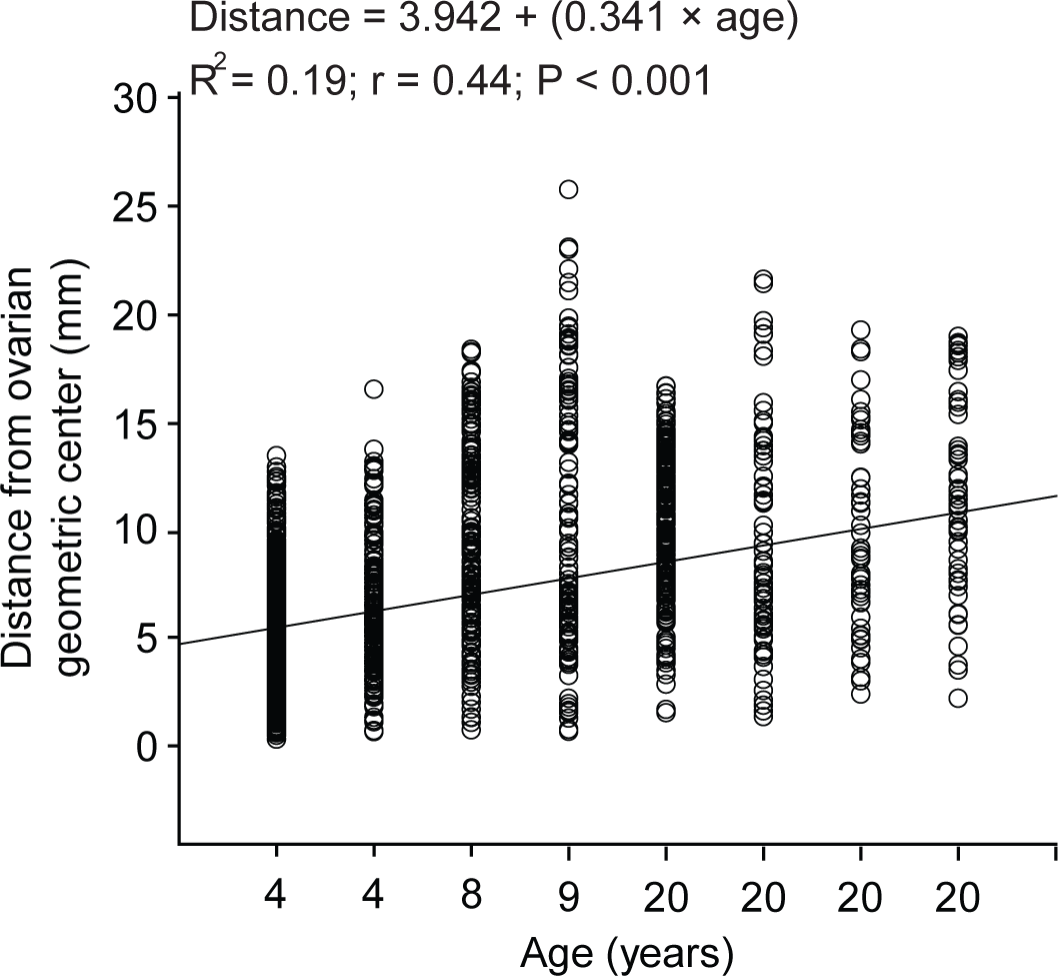
Age effect on the mean distance of equine preantral follicles to the ovarian geometric center recorded in histological sections (n = 240). Each circle of the graph represents an evaluated preantral follicle (n = 9,284). Linear regression and correlation analyses are presented.

The mare age influenced the mean distance from preantral follicles to the geometric center (Fig 3; Table 4). A regression analysis showed that the distance of preantral follicles to the ovarian geometric center was positively (*P* < 0.001) associated with age (Fig 3). Young mares had preantral follicles located closer (*P* < 0.05) to the geometric center in all evaluated ovarian portions and regions compared to old mares (Table 4). Moreover, regardless of age group and ovarian portion, preantral follicles in the ventral region were located farther (*P* < 0.05) from the geometric center than those observed in the dorsal region.

**Table 4:** Age effect on mean distance (± SEM) from equine preantral follicles to the geometric center according to different ovarian portions (lateral and intermediary) and regions (dorsal and ventral).

	Distance from preantral follicles to ovarian geometric center (mm)					
	Ovarian portion^§^					
	Lateral		Intermediary		Overall	
	Age group (years)					
Ovarian region^‡^	4–9	>20	4–9	>20	4–9	>20
Dorsal (n = 4,901)^†^	5.42 ± 0.04^a^^A^	8.41 ± 0.41^b^^A^	4.62 ± 0.04^a^^A^	9.64 ± 0.23^b^^A^	5.05 ± 0.03^a^^A^	9.31 ± 0.20^b^^A^
Ventral (n = 4,383)	5.47 ± 0.07^a^^B^	10.19 ± 0.47^b^^B^	6.58 ± 0.06^a^^B^	11.56 ± 0.24^b^^B^	5.97 ± 0.05^a^^B^	10.87 ± 0.23^b^^B^
Overall	5.44 ± 0.04^a^	9.27 ± 0.32^b^	5.45 ± 0.04^a^	10.31 ± 0.17^b^	5.44 ± 0.03^a^	10.01 ± 0.15^b^

^§^ The ovaries were divided longitudinally into three portions: two laterals and one intermediary. Both ovaries (left and right) from each mare (n = 8) were used.

^‡^ Ovarian regions were defined as dorsal (above) and ventral (below) according to the geometric center and ovulation fossa.

^†^ Number of preantral follicles evaluated per ovarian region.

^a,b^ Within the same ovarian portion and between age groups, values without a common superscript differed (*P* < 0.05).

^A,B^ Within a column, values without a common superscript differed (*P* < 0.05).

**Fig 4:**
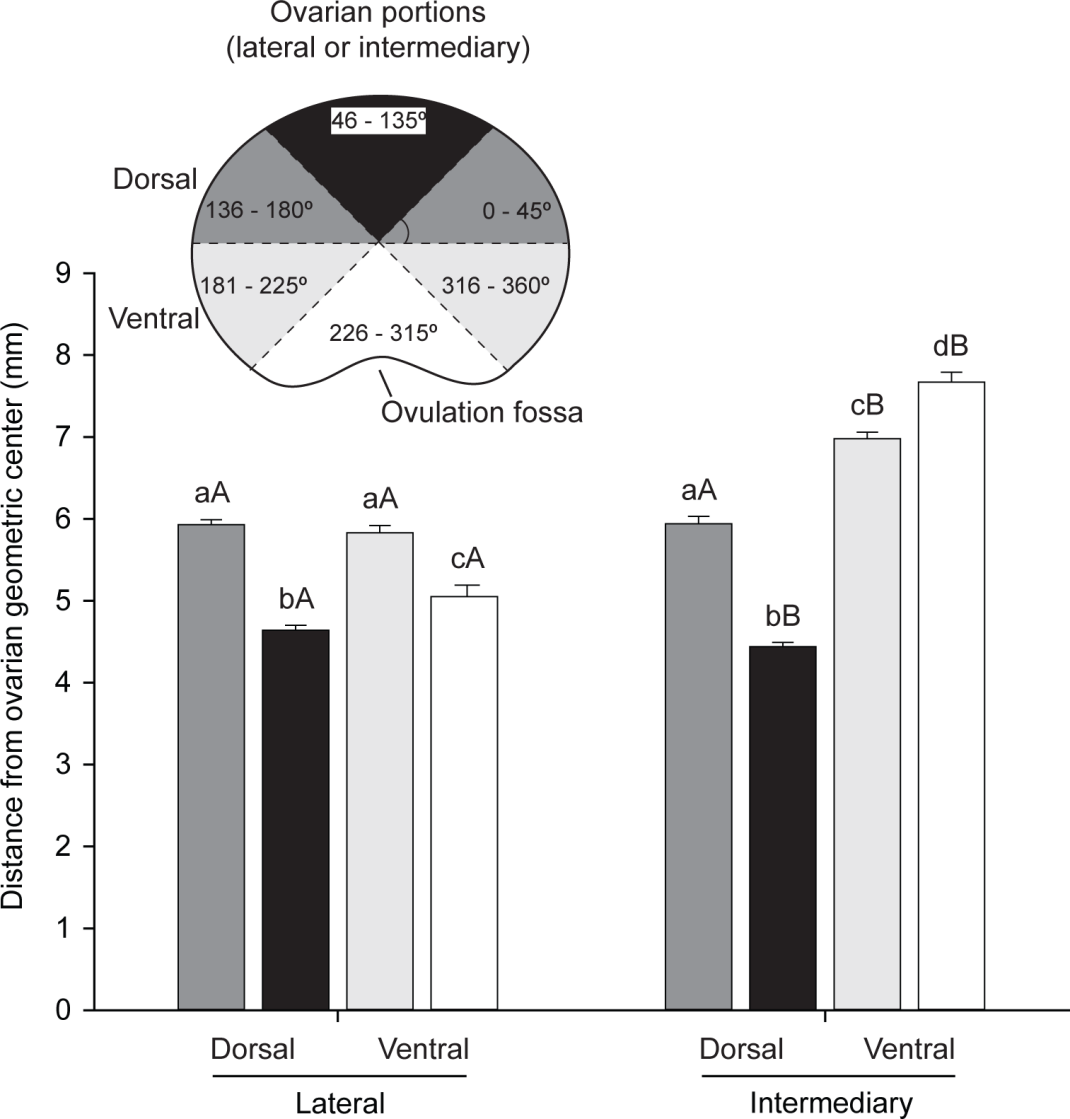
Mean (± SEM) distance of equine preantral follicles from ovarian geometric center according to angle position in different ovarian portions (lateral and intermediary) and regions (dorsal and ventral). ^a,b,c,d^ Indicate difference within the same ovarian portion (lateral or intermediary) evaluated (*P* < 0.05). ^A,B^ Differ between ovarian portions within the same angle position assessed (*P* < 0.05).

To study the spatial distribution of preantral follicles in the equine ovary, the distance of preantral follicles from the geometric center according to their angular position intervals was evaluated for each ovarian region (Fig 4). Within the dorsal region at angular position of 46º˗135º, the preantral follicles were closer (*P* < 0.05) to the geometric center in both lateral and intermediary portions compared to the ventral region. In addition, the preantral follicles located in the ventral region of the intermediary portion at angular positions of 181º˗225º, 226º˗315º, and 316º˗360º were farther (*P* < 0.05) from the geometric center in comparison to similar angular positions of the lateral portion.

**Fig 5:**
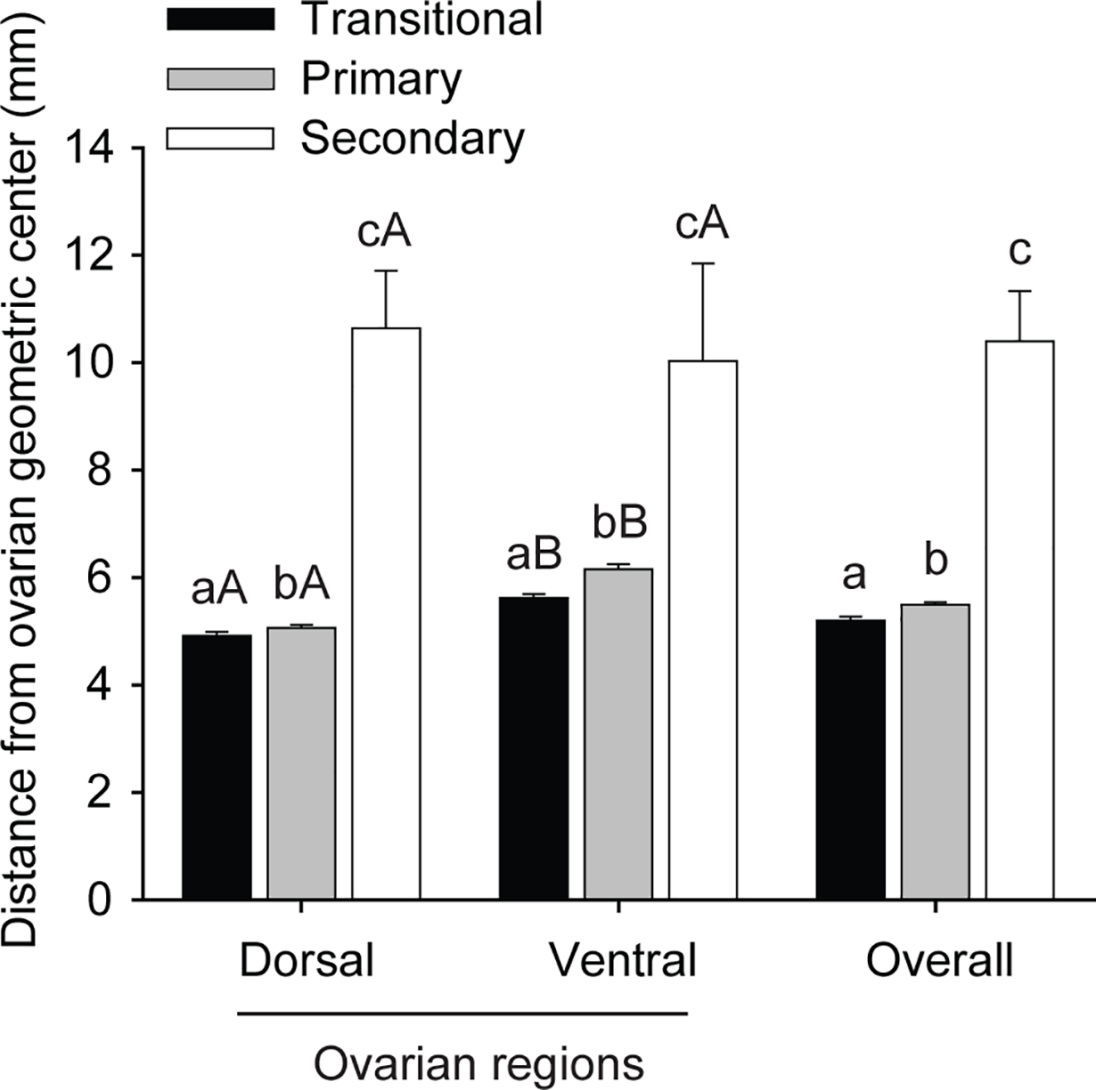
Mean (± SEM) distance of developing follicles (transitional, primary, secondary) from geometric center in different ovarian regions (dorsal and ventral). ^a,b,c^ Indicate difference within the same ovarian region (ventral or dorsal; *P* < 0.05). ^A,B^ Differ between ovarian regions within the same follicular category (*P* < 0.05).

Regarding follicular classes, the distances from primordial and developing follicles to the geometric center according to different ovarian portions and regions are shown (Table 5). In general, primordial and developing follicles in the ventral region were located farther (*P* < 0.05) from the ovarian geometric center than those in the dorsal region. The developing follicles were closer (*P* < 0.05; overall results) to the geometric center compared with primordial follicles, regardless of ovarian portions and regions. When considering the developing follicles only (Fig 5), the distance from the geometric center increased (*P* < 0.05) as the follicles developed from transitional to secondary follicle category in either the dorsal or ventral ovarian regions. Also, the transitional and primary follicles located in the ventral region were farther (*P* < 0.05) from the geometric center compared to the same stage follicles evaluated in the dorsal region.

**Table 5:** Mean (± SEM) distance (mm) from primordial and developing follicles (transitional and primary) to the ovarian geometric center according to different ovarian portions (lateral and intermediary) and regions (dorsal and ventral).

	Distance from preantral follicles to ovarian geometric center (mm)					
	Ovarian portion^§^					
	Lateral		Intermediary		Overall	
Ovarian region^‡^	Primordial	Developing	Primordial	Developing	Primordial	Developing
	(n = 1,931)^†^	(n = 2,962)	(n = 1,660)	(n = 2,716)	(n = 3,591)	(n = 5,678)
Dorsal	5.77 ± 0.10^a^^A^	5.35 ± 0.05^a^^A^	6.06 ± 0.11^a^^A^	4.55 ± 0.04^b^^A^	5.91 ± 0.07^a^^A^	4.98 ± 0.03^b^^A^
	(0.6–22.1) ^¥^	(0.6–23.0)	(0.3–18.7)	(0.3–18.9)	(0.3–22.1)	(0.3–23.0)
Ventral	6.00 ± 0.13^a^^A^	5.39 ± 0.09^b^^B^	8.25 ± 0.14^a^^B^	6.24 ± 0.06^b^^B^	7.03 ± 0.09^a^^B^	5.80 ± 0.06^b^^B^
	(0.4–23.1)	(0.4–25.7)	(0.5–19.7)	(0.5–19.1)	(0.4–23.1)	(0.4–25.7)
Overall	5.87 ± 0.08^a^	5.37 ± 0.05^b^	7.05 ± 0.09^a^	5.24 ± 0.04^b^	6.42 ± 0.06^a^	5.31 ± 0.03^b^
	(0.4–23.1)	(0.4–25.7)	(0.3–19.7)	(0.3–19.1)	(0.3–23.1)	(0.3–25.7)

^§^ The ovaries were divided longitudinally into three portions: two laterals and one intermediary. Both ovaries (left and right) from each mare (n = 8) were used.

^‡^ Ovarian regions were defined as dorsal (above) and ventral (below) according to the geometric center and ovulation fossa.

^†^ Number of primordial and developing follicles (transitional and primary) evaluated per ovarian portion.

^¥^ Range of follicles.

^a,b^ Within the same ovarian portion (lateral or intermediary) and between follicular class, values without a common letter differed (*P* < 0.05).

^A,B^ Within a column, values without a common letter differed (*P* < 0.05).

**Fig 6:**
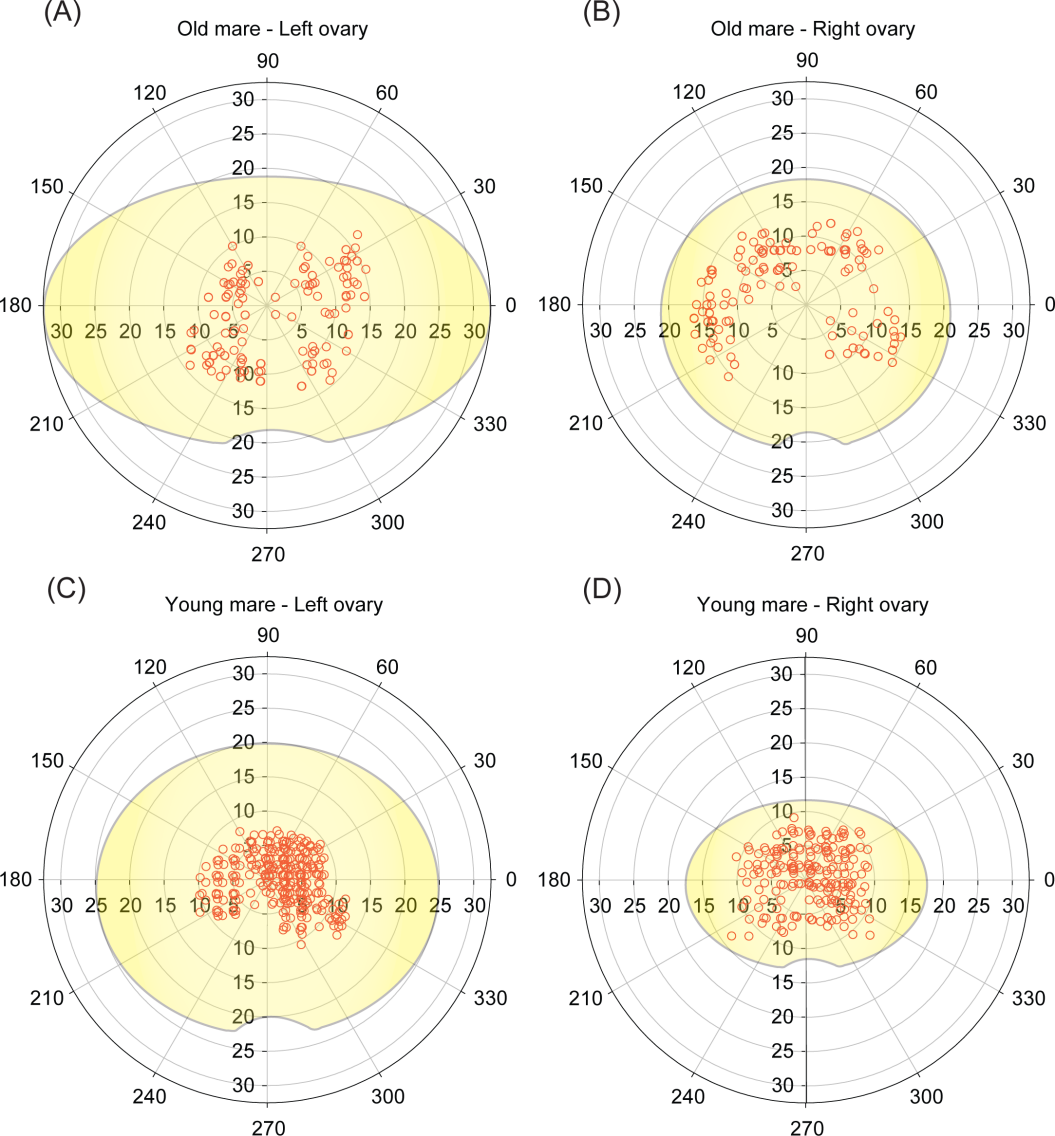
**Polar coordinate plots of representative mares showing the spatial distribution of preantral follicles (combined ovarian portions) on equine ovarian cortex according to age [(A,B) old mare; (C,D) young mare] and side ovary [(A,C) left ovaries; (B,D) right ovaries].** Each preantral follicle was represented by an open red circle. The spatial distribution was determined by its angulation (0º˗360º) and distance (mm) in relation to ovarian geometric center. The drawn ovaries (yellow) represent the original dimensions (length and height) from each animal.

**Fig 7:**
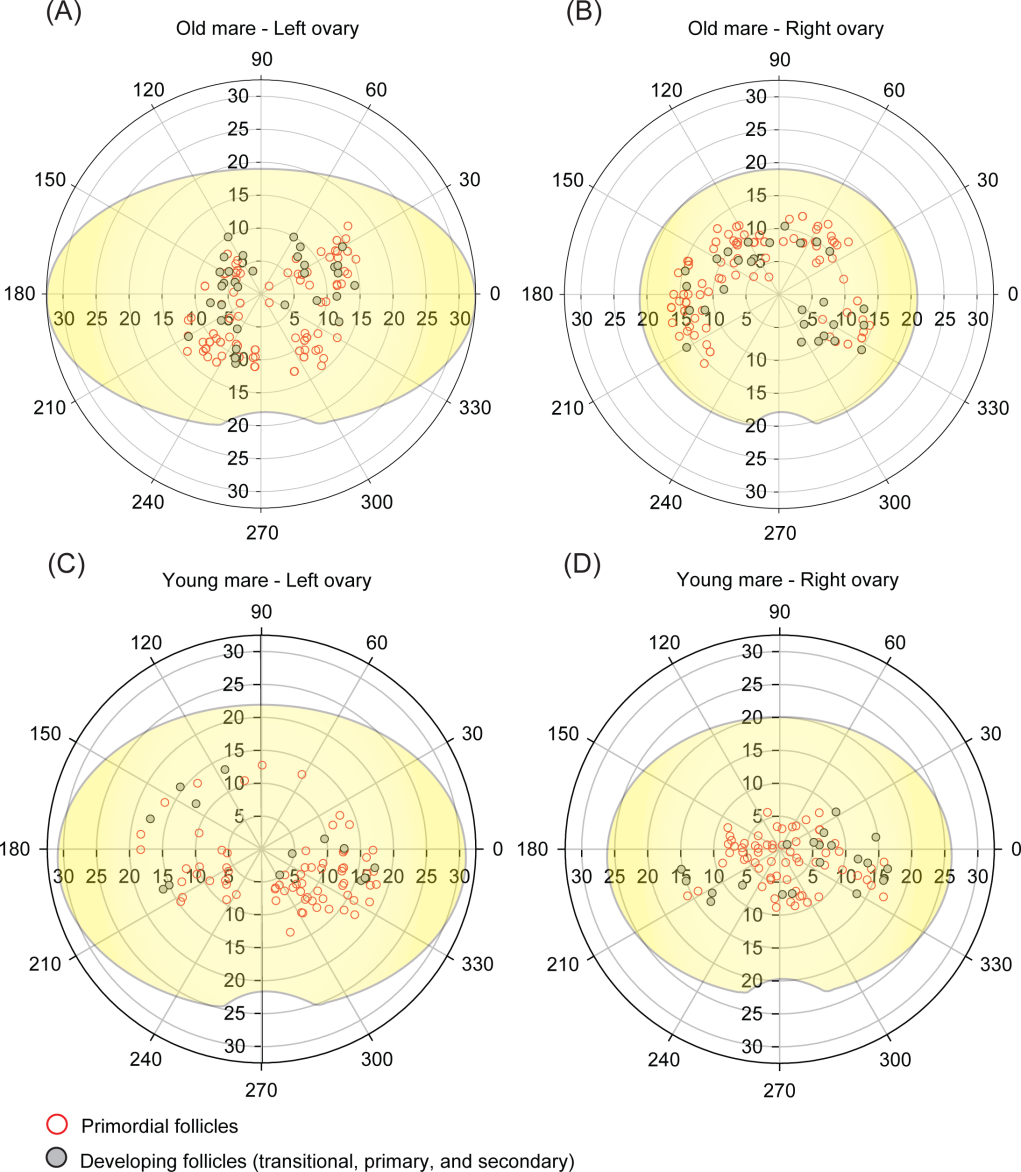
**Polar coordinate plots of representative mares showing the spatial distribution of primordial and developing follicles (combined ovarian portions) on equine ovarian cortex according to age [(A,B) old mare; (C,D) young mare] and side ovary [(A,C) left ovaries; (B,D) right ovaries].** Preantral follicles were represented by filled gray and open red circles. The spatial distribution was determined by its angulation (0º˗360º) and distance (mm) in relation to ovarian geometric center. The drawn ovaries (yellow) represent the original dimensions (length and height) from each animal.

**Fig 8:**
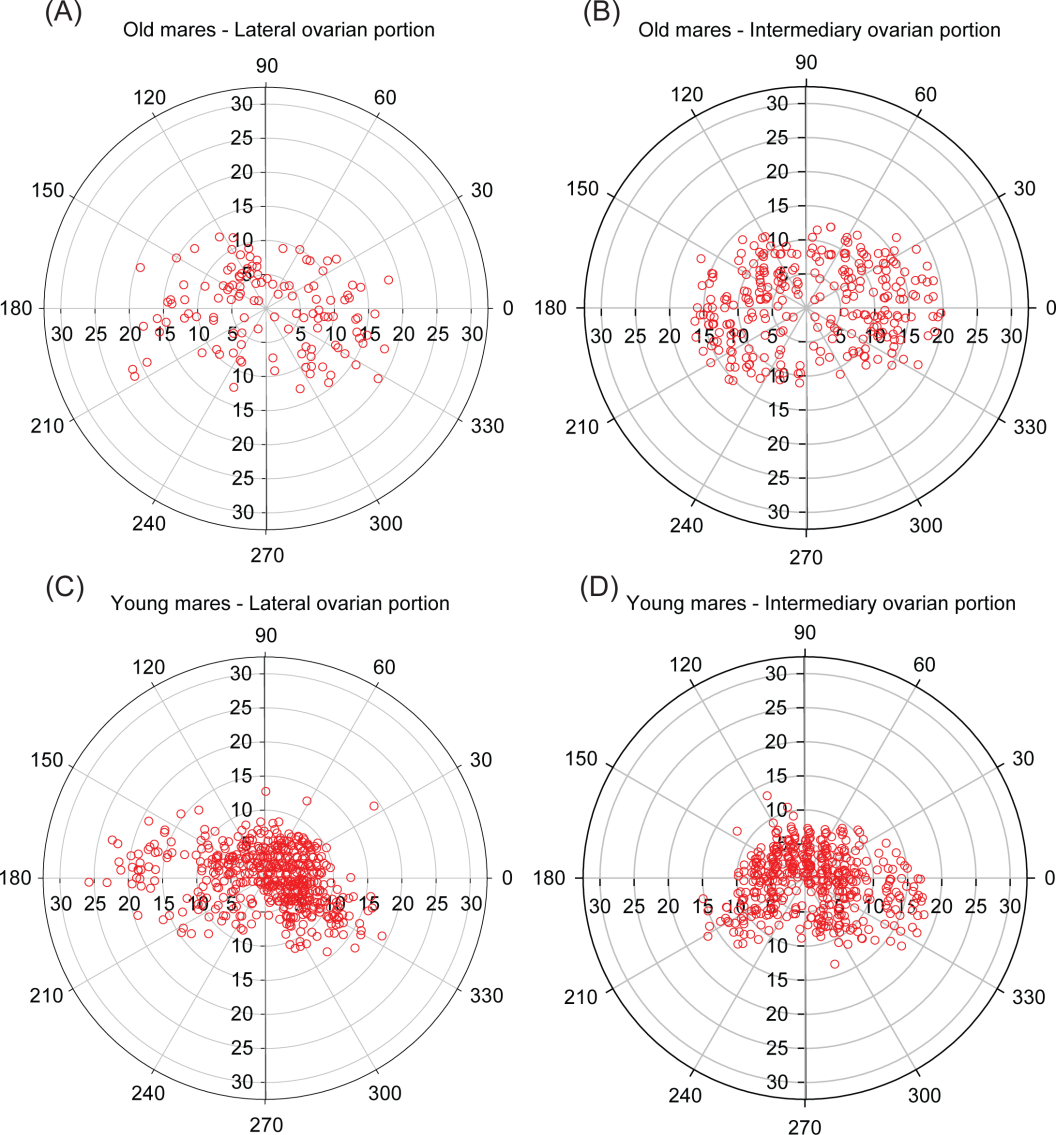
**Polar coordinate plots showing the spatial distribution of preantral follicles according to age [(A,B) old mares, n = 4; (C,D) young mares, n = 4] and ovarian portions [(A,C) lateral; (B,D) intermediary].** Preantral follicles (combined data per mare within each age group) were represented by open red circles. The spatial distribution of each preantral follicle was determined by its angulation (0º˗360º) and distance (mm) in relation to ovarian geometric center.

Based on all previous analyses (frequency distribution, distance from the geometric center, and angle position), the spatial distribution of equine preantral follicles considering the effect of age (Fig 6), follicular class (Fig 7), and ovarian portions (Fig 8) has been demonstrated using schematic illustrations (polar coordinate plots) with the original ovarian dimensions (length and height) of representative mares.

## Discussion

The present study developed a novel methodology for objective and accurate assessment of the preantral follicle spatial distribution in the equine ovarian parenchyma using histology, computational image analysis, and mathematical calculations. Furthermore, the influence of age on preantral follicle spatial distribution was observed. Results herein described have the potential to be translated to animals of the equidae family, and to advance our knowledge towards characterization of mechanisms involved in ovarian plasticity [28] with migration of preantral follicles throughout the ovarian parenchyma during the early phases of folliculogenesis.

In the present study, a crowding effect of preantral follicles closer to the geometric center (circle interval of <10 mm radius) was observed in young mares, demonstrating that as the mare ages, the spatial follicle distribution changes to a more dispersive pattern in the ovarian cortex. These results support recent findings from our studies where a higher variability and an age effect on the number and density of preantral follicles in the equine ovarian tissue were observed [10,13–15,24]. Similar results have been reported in the mouse where aging was associated with a less homogeneous preantral follicle spatial distribution in the ovary because of increasing clustering of follicles [1]. The formation, proliferation, and colonization of primordial germ cells on ovarian parenchyma are regulated by genes [29] and factors [30], and may be species specific.

This work showed important differences in preantral follicle spatial distribution between dorsal and ventral regions and between lateral and intermediary portions. Furthermore, the distance of preantral follicles in those regions also differed from the geometric center. In mammals, preantral follicles are not evenly distributed throughout the ovarian cortex, and a great follicular heterogeneity has been observed among and within individuals (woman [31]; cow [32]; mare [24]; sheep [19]). Heterogeneity of preantral follicle distribution in the equine ovary among fragment samples (1.5 × 0.5 × 0.5 cm) collected from the innermost, middle, and outermost regions was recently reported [12]. In addition, the former authors reported a greater preantral follicle population near rather than far from the ovulation fossa. Comparisons between the results from the present study with those of Gonzalez et al. [12] are not feasible due to the huge differences between the experimental designs.

Knowledge regarding the spatial distribution of preantral follicles in the ovarian cortex might be useful to guide ovarian biopsy procedures and improve the recovery rate of ovarian fragments with a satisfactory number and density of preantral follicles. Furthermore, this knowledge will allow the biopsy procedures to reduce: (i) the number of punctures per ovary; (ii) number of ovarian fragments to be collected; (iii) the number of animals in experimentation; and (iv) the time to perform the procedure. Results of this study highlight the importance of considering the ovarian regions and age of the animals during biopsy procedures. Therefore, taking into account the difference between young and old mares in terms of preantral follicle spatial distribution from the geometric center, it is recommended for a better success (fragment with a richer preantral follicle population) of the biopsy procedure to target the center of the ovary in young mares and more distant areas from the geometric center in old mares.

Our novel findings also showed different distance patterns from the geometric center among primordial and developing follicles regardless of ovarian portions and regions. The results demonstrated that initially after follicle activation the distance from the geometric center reduced, but then increased after secondary follicles developed. A recent report [2] showed an inhibitory effect of resting follicle crowding on the probability of neighboring follicles activation. Indeed, this behavior seems to be crucial to prevent the premature recruitment and consequent exhaustion of the ovarian follicle reserve. Mechanical factors such as physical forces (tension and compression), remodeling of extracellular matrix, and changes in cell shape modify biochemical mediators that induce changes on the transcriptional activities and alter cell form and function [4,33]. In mice, during the growth process, preantral follicles migrate from the cortex towards to medulla, which has a softer and more pliant extracellular matrix [28]. In horses, extensive tissue remodeling is required for antral follicle growth and migration to the ovulation fossa [3].

In the present study, secondary follicles located in the ventral region of the ovary were farther from the geometric center but closer to the ovulation fossa. These follicles will later give rise to the early tertiary (antral) follicles. It is well known that antral follicles are distributed randomly in the cortical area of the equine ovary [7–9]; however, only the ovulatory follicles migrate towards the ovulation fossa to be able to ovulate. Based on the findings of this study related to preantral follicle spatial distribution in relation to the geometric center, the following parts of a working hypothesis have been proposed. Initially, primordial follicles are located farther than developing follicles from the geometric center (i.e., center of the ovary). Then, after primordial follicle activation/recruitment, the transitional and primary follicles migrate towards the geometric center. However, as follicle development continues, secondary follicles migrate to areas farther from the geometric center to later form antral follicles.

In conclusion, the spatial distribution of preantral follicles was successfully determined in the equine ovary and was influenced by age, region, and portion. The information on spatial distribution of preantral follicles in the equine ovary gained in this study can be helpful for targeting areas with greater numbers of follicles in young and old mares (areas closer to and farther from the geometric center, respectively), improving as a result the harvesting of preantral follicles through the biopsy procedure. Furthermore, the insights herein obtained regarding spatial distribution of preantral follicles may contribute to elucidate mechanisms related to follicular quiescence and activation, cell-cell and cell-extracellular matrix interactions, follicular migration, and ovarian plasticity.

## Supporting information

S1 Table (XLS)
